# CMS AND ASTP: FEDERAL PARTNERSHIPS FORGED TO SUPPORT THE DEVELOPMENT OF INTEROPERABLE E-CARE PLANS

**DOI:** 10.1093/geroni/igae098.4260

**Published:** 2024-12-31

**Authors:** Elizabeth Palena Hall, Michael Wittie

**Affiliations:** The Centers for Medicare and Medicaid Services, Baltimore, Maryland, United States; Assistant Secretary for Technology Policy and Office of the National Coordinator for Health Information Technology (ASTP/ONC), Washington, District of Columbia, United States

## Abstract

Across the United States, more than 25% of Americans have multiple chronic conditions that often impact quality of life, mortality, complexity of care and cost. The need for seamless interdisciplinary communication and data driven decision-making continues to grow, however, health care often remains fragmented, poorly coordinated and inefficient. A comprehensive, shared, interoperable e-care plan has the potential to improve patient outcomes, satisfaction, and quality of care by making comprehensive health data available across providers and settings when and where it is needed most. At the Federal level, the Centers for Medicare & Medicaid Services (CMS) and the Assistant Secretary for Technology Policy/Office of the National Coordinator for Health IT (ASTP) are engaged in efforts to improve interoperable e-care plans. This session will describe the current state of e-care plans and challenges, promising practices from the field, and opportunities for advancement. This presentation will discuss how ASTP programs interact with CMS’ work to support an e-care plan environment where patients’ care plan information is securely and readily available to support patient outcomes. The session will include an overview of updates and proposals to ASTP’s Health IT Certification Program, a description of how the United States Core Data for Interoperability (USCDI) and USCDI+ Program can support the collection and exchange of e-care plans with the care team and patients, support quality measurement and quality reporting programs, and highlight how interested parties can engage in ongoing standards and policy development.

